# Violence exposure and young people’s vulnerability, mental and physical health

**DOI:** 10.1007/s00038-020-01340-3

**Published:** 2020-02-18

**Authors:** Andrew Clarke, Philippa Olive, Naseerah Akooji, Karen Whittaker

**Affiliations:** 1 Lancashire Care NHS Foundation Trust, Preston, UK; 2 SEaRCH (Supporting Evaluation and Research in Child and Family Health) Research Group, School of Nursing, University of Central Lancashire, Preston, UK; 3 Lancashire Clinical Trials Unit, Faculty of Health and Wellbeing, University of Central Lancashire, Preston, UK; 4 Save the Children UK, London, UK

**Keywords:** Domestic violence, Relationship violence, Young people, Adolescence, Health, Well-being

## Abstract

**Objectives:**

To analyse the impact of being affected by domestic and/or relationship violence in early adolescence on indicators of health and well-being.

**Methods:**

Secondary data analysis of a cross-sectional survey of 13–14 year-old pupils attending schools in north-west England, with variables relating to vulnerability, violence and mental and physical health, was performed. The sample of 9626 represented 71% of the eligible population. Chi-squared tests and logistic regression were used to analyse demographic exposure to violence and outcomes.

**Results:**

Pupils affected by domestic and/or relationship violence had significantly worse outcomes and experiences than non-affected peers. Odds ratios demonstrated higher risks of being lonely, being bullied or having deliberately self-harmed. They were also more likely to report an enduring health condition, poorer health practices and worse access to and experiences of health services.

**Conclusions:**

Exposure to violence in domestic and/or relationships is detrimental to children and young people’s mental and physical health and vulnerability. Health risks and inequalities reported by CYP in this study provide compelling intelligence for renewed strategic policy-level consideration in the design and delivery of young peoples’ health services.

## Introduction

Exposure to violence, as a witness to domestic violence within the family or the subject of direct violence perpetrated within other relationships, is a human rights violation and a damaging global phenomenon experienced by many children and young people (CYP) (UN 1989; UNICEF 2012). Addressing violence is a public health priority reflected in global (UN 2015), regional (WHO 2014) and national (HMG 2016) health and protection strategies and policies. There are a range of terms for domestic and relationship violence used across this field. Therefore, to acknowledge and clearly identify these differences, when referring to other research, their term was used so as not to infer equivalence across studies.

Surveys in USA and Europe found 12% of CYP reported being direct victims of physical and/or psychological violence at home in the last year (Mrug and Windle 2010) and 24% reported lifetime prevalence (Kassis et al. 2013). Witnessing inter-parental violence in the last year was reported by 12% of CYP (Mrug and Windle 2010) and lifetime witnessing ranged from 10 (Sprah 2008) to 17% (Kassis et al. 2013). Physical violence, psychological and emotional violence and sexual violence in CYP’s dating relationships were reported as between 10 and 30%, 35 and 55% and 5 and 30%, respectively (Stonard et al. 2014). Rate of dating violence increased with young people’s age (Coker et al. 2014) and was greater for those identifying as lesbian, gay and bisexual (Peters et al. 2017).

CYP’s exposure to violence by adults at home, either through witnessing or victimisation, had medium effect sizes (range 0.51–0.74) for anxiety, symptoms of depression and lower self-esteem, compared to CYP not exposed (Gunnlaugsson et al. 2011). Similarly, CYP’s exposure to violence in their own relationships has been associated with increased rates of anxiety and depressive symptoms (Barter and Stanley 2016). In a meta-analysis, the effect size for trauma symptoms (flashbacks, hyperarousal and withdrawal) amongst CYP exposed to violence by adults at home, either through witnessing or victimisation, compared to non-exposed was 1.58 (Davies et al. 2008). Suicidal ideation amongst CYP that witnessed family violence in the previous year was 11.4% compared to 3.5% amongst non-witnessing CYP (*p* < 0.001) (Turner et al. 2012). CYP exposed to parental violence are more likely to be a victim and perpetrator of bullying than non-exposed peers. Odds ratios for bullying victimisation ranged from 2.6 (witnessing) to 20.3 (victimisation) and from 1.8 (witnessing) to 17 (victimisation), and risk increased with frequency and severity (Lucas et al. 2016). CYP reporting being bullied or cyber-bullied were also more likely to be victimised in their own relationship (ORs 2.5 and 3.0, respectively) (Peters et al. 2017).

CYP who witnessed parental partner violence were also twice as likely to report poorer general health (Helweg-Larsen et al. 2011) and more likely to have higher body mass index (Gooding et al. 2015). Young women exposed to dating violence were more likely to suffer eating disorders and sleep disturbances (Barter and Stanley 2016) and to report greater physical ailments (Haynie et al. 2013). CYP who witnessed or were victims of family violence reported feeling undeserving of attention, affection and suspicious of others’ intentions (Calvete et al. 2018). Similarly, young adults who witnessed parental violence were distrustful, felt unsafe (van Rosmalen-Nooijens et al. 2017) and less likely to seek professional help (Lepistö et al. 2010).

The data source for this study is an annual school health needs assessment undertaken in the north-west of England. This is a unique data set as neither schools nor health services in the UK are required to undertake health needs assessments of this type or coverage. The data are primarily collected to inform service provision and local public health policy across localities and within individual schools and supports clinical care to individual pupils. In this study, CYP’s potential vulnerability from violence in their domestic (family) and dating relationships was collapsed into one category and termed ‘domestic and/or relationship violence’.

Despite not primarily being a research tool, the breadth of topics covered in the survey allows for a wider range of health and well-being outcomes to be analysed within the same data set than is usually possible. We have maximised the potential of these data, recognising that data collected specifically for research purposes would have allowed more flexibility and rigour in the analysis.

The expanded hypothesis for this study was that in addition to known associations between domestic and/or relationship violence and mental health, physical health and health risk behaviours, the presence of domestic and/or relationship violence may be a proxy indicator for a wider set of health vulnerabilities connected with psychological and physiological stress responses (WHO 2013), family disruption or sub-optimal care-giving environments (Shonkoff and Garner 2011). Therefore, it was conducted without predetermining selected variables to identify the spectrum of health and well-being risk experienced by CYP affected by domestic and/or relationship violence, and spoke to the corresponding research question: What are the mental and physical health and vulnerability risks experienced by CYP in Lancashire affected by domestic and/or relationship violence?

## Methods

A secondary data analysis of the School Health Needs Assessment (SHNA) cross-sectional survey of Year 9 (13–14 years) pupils attending secondary schools in two local authorities in north-west England was performed.

### The SHNA survey

The annual SHNA exercise, implemented across two local authorities by school nursing teams since 2011, included all primary and secondary schools covered by Lancashire Care NHS Foundation Trust (approximately 500). It is designed to assess and respond to the individual and collective health rights and needs of CYP. The process draws together data and intelligence from existing public health sources, school leaderships and CYP. The collated intelligence provides a meaningful understanding of health needs and priorities in each school community. Anonymised survey data, aggregated to larger populations, are used to inform service design, public health planning, policymaking and decision-making.

### Sample

All pupils in Year 9 (13–14 years) attending government high schools covered by health services from Lancashire Care NHS Trust were considered eligible. The sampling frame was 13,557 pupils, and surveys undertaken between September 2016 and December 2017 were included for analysis.

### Recruitment

School nurses attended special assemblies to explain verbally and via written, age-appropriate information about the purpose of the survey. Pupil’s participation was voluntary, with reassurance of no penalty for non-participation. Pupils were assured that individual information would not be shared with school without their permission and would be held in their confidential health service records; but that anonymised data would be shared with schools and public health planners.

### Data collection

The survey was initially constructed as a composite tool using questions drawn from validated questionnaires such as the Global Student School-based Health Survey (WHO 2003), the WHO Multi-country Study on Women’s Health and Domestic Violence against Women (WHO 2005), the Strengths and Difficulties Questionnaire (Goodman et al. 1998) and tools used commonly by non-governmental organisations. Local testing and contextualisation of new questions were undertaken where existing and appropriate questions could not be found. These were matched to fields of enquiry determined by domains of the UK Government’s Healthy Child Programme (DH 2009), Every Child Matters agenda (DfES 2003) and priorities of school-age service users. Survey tools and processes have been subject to annual multi-stakeholder review for the previous 6 years, resulting in further refinements and revisions in framing and content. To optimise the tool for readability, advice was sought with regards to the format, paper colour, type face and font size, to reduce barriers for CYP with dyslexia.

The self-reporting survey questionnaire collected named data about gender, ethnicity, sexual and gender identity, with thirty questions (49 items—38 binary, 4 Likert scale, 2 multiple choice and 5 open text) about mental and physical health, access and experience of health care, healthy and risk behaviours, vulnerabilities and sexual and reproductive health. Sociodemographic data about family structure or socioeconomic status were not collected. Question 17 of the survey (‘Have you ever been affected by domestic abuse/violence (including physical or emotional abuse) in your family or other relationships?’) is the predictor variable of interest for this analysis.

School nurses distributed the survey in classrooms and remained during completion to address any questions about participation or survey questions. Completed surveys were returned to school nurse offices where they were reviewed to identify pupils requiring follow-up, and the data were entered into pupil’s health records. Anonymised data were entered into a web-based database and collated in a password-protected Excel spreadsheet.

### Data analysis

The data set was exported and analysed in IBM SPSS (version 24.0). Frequencies for demographic variables were obtained, and chi-squared tests were used to ascertain whether CYP’s report of being affected by domestic and/or relationship violence differed between demographic groups (Table 1). Logistic regression was used for analyses of all outcomes, with CYP’s report of being affected by domestic and/or relationship violence as the predictor variable of interest and regression outcomes defined as a positive or a negative health outcome. (Negative outcomes indicators are those that would be considered to have a negative impact on CYP’s health and well-being; Table 2.) Each regression was adjusted for gender and ethnicity separately, as well as allowing for possible statistical interaction between gender and ethnic group. Sensitivity analysis to assess whether missing survey answers relating to domestic and/or relationship violence affected the findings concluded them to be missing at random, and those estimates of odds ratios obtained in the main analysis have little bias.

**Table 1:** Demographic frequencies of young people participating in Lancashire School Health Needs Assessment survey, England, September 2016 to December 2017

	% Affected by domestic and/or relationship violence	*n* in category^a^	Chi-squared test
Male	5.6	4623	*χ*^2^(2) = 20.97 *p *< 0.001
Female	7.8	4527	
Other gender	15.4	26	
Total		9176	
Cisgender^b^	6.5	6352	*χ*^2^(1) = 7.70 *p *= 0.006
Non-cisgender	13.3	105	
Total		6457	
White	7.4	6560	*χ*^2^(3) = 27.35 *p *< 0.001
Mixed	9.0	289	
Asian or Asian British	4.5	2246	
Black or Black British	10.3	68	
Total		9163	
Straight	6.2	8628	*χ*^2^(3) = 89.39 *p *< 0.001
Gay/lesbian	11.7	60	
Bisexual	21.1	209	
Other	15.3	118	
Total		9015	

^a^9273 reported information for the violence variable; the category total reflects the missing values within that category

^b^Cisgender: a person who self-identifies their gender as that of their birth sex

**Table 2:** Frequencies and odds ratios with 95% confidence intervals for the associations between domestic and/or relationship violence and the mental and physical health and vulnerabilities of young people affected by domestic and/or relationship violence participating in Lancashire School Health Needs Assessment survey from, England, September 2016 to December 2017

Lancashire School Health Needs Assessment survey questions collated by domains	Response for analysis	Exposed to domestic and/or relationship violence		Not exposed to domestic and/or relationship violence		Odds ratio	95% confidence interval	*p* value
		Total *N*	% (*n*) with outcome	Total *N*	% (*n*) with outcome			
*Mental health*								
Do you often feel happy?	No	580	28 (160)	8287	6 (459)	6.02	4.88, 7.46	< 0.001
Do you often feel hopeful?	No	573	34 (197)	7869	14 (1093)	3.02	2.50, 3.65	< 0.001
Do you often feel angry?	Yes	571	66 (376)	7773	35 (2696)	3.50	2.92, 4.20	< 0.001
Do you often feel lonely?	Yes	578	45 (259)	7755	14 (1073)	4.85	4.03, 5.85	< 0.001
About your weight, do you think you are…(negative perception of weight)	Underweight/overweight	608	42 (258)	8378	26 (2135)	2.22	1.87, 2.63	< 0.001
Have you been bullied more than once in the last 2 months?	Yes	616	28 (172)	8542	9 (759)	3.76	3.10, 4.57	< 0.001
Have you been cyber-bullied (someone sending mean instant messages, wall-postings, emails and text messages)?	Yes	616	32 (197)	8491	10 (842)	4.00	3.30, 4.83	< 0.001
Do you know where to get help about bullying?	No	618	12 (75)	8577	6 (522)	2.13	1.64, 2.78	< 0.001
Have you taken part in bullying someone else during the last 2 months?	Yes	611	10 (59)	8588	3 (250)	3.80	2.81, 5.15	< 0.001
Have you ever hurt or harmed yourself deliberately?	Yes	611	36 (219)	8551	8 (692)	5.88	4.88, 7.09	< 0.001
*Physical health*								
Do you have any illnesses, conditions, or attend any regular hospital clinics, have regular health support or take regular medicine?	Yes	581	25 (146)	8065	17 (1342)	1.65	1.35, 2.01	< 0.001
Do you go to the dentist?	No	622	6 (37)	8607	3 (241)	2.48	1.72, 3.56	< 0.001
Did you brush your teeth two times yesterday?	No	622	15 (93)	8606	8 (700)	2.19	1.71, 2.79	< 0.001
Yesterday, did you eat something for breakfast, dinner and tea?	No	622	40 (248)	8587	20 (1698)	2.53	2.13, 3.01	< 0.001
Did you eat any fruit or vegetables yesterday?	No	616	21 (131)	8530	16 (1372)	1.51	1.23, 1.86	< 0.001
How often do you drink sugary soft drinks?^1^	Every day/more than once a day	618	40 (243)	8496	31 (2618)	1.51	1.27, 1.80	< 0.001
On a week day how many hours do you usually spend sitting or lying down watching television, videos, computer games? (sedentary behaviour)	2–3 h/more than 3 h	619	66 (410)	8538	57 (4866)	1.53	1.28, 1.82	< 0.001
Have you ever smoked cigarettes?	Yes	605	25 (150)	8463	5 (456)	5.56	4.48, 6.85	< 0.001
Have you ever smoked E-cigarettes?	Yes	612	34 (211)	8495	11 (951)	4.15	3.44, 4.98	< 0.001
Have you ever smoked shisha?	Yes	595	10 (62)	8431	4 (332)	3.27	2.42, 4.42	< 0.001
Have you ever been drunk?	Yes	620	27 (165)	8509	9 (744)	3.52	2.87, 4.31	< 0.001
*Vulnerability*								
Are you worried about anyone close to you who uses drugs or alcohol?	Yes	615	22 (136)	8430	6 (533)	1.88	1.38, 2.54	< 0.001
During the last year have you been worried about drug use in your school or in your group of friends?	Yes	597	9 (54)	8230	5 (394)	3.98	3.22, 4.95	< 0.001
Have anyone ever asked you to try drugs?	Yes	613	30 (180)	8477	7 (609)	5.49	4.50, 6.71	< 0.001
Would you feel confident to say no if someone wanted to have physical or intimate contact with you and you didn’t want to?	No	595	16 (93)	8262	10 (789)	1.96	1.54, 2.49	< 0.001
Do you think you have enough knowledge to know how to prevent unwanted pregnancy?	No	616	20 (121)	8294	17 (1395)	1.40	1.13, 1.74	< 0.001
Do you think you have enough knowledge to know how to prevent an infection you can get from sex?	No	617	31 (191)	8269	28 (2328)	1.27	1.05, 1.53	< 0.001
Are you helping to look after someone at home or in your family?	Yes often/everyday	628	21 (133)	8645	12 (1018)	2.18	1.78, 2.69	< 0.001
Do you have an adult you can talk to about any problems?	No	620	17 (103)	8532	6 (519)	3.23	2.55, 4.08	< 0.001
Do you know how to get information about your health if you want to?	No	607	20 (120)	8348	17 (1119)	1.63	1.32, 2.01	< 0.001
The last time you went to see a health professional, were you given enough information?	No	613	17 (101)	8383	7 (577)	2.69	2.13, 3.40	< 0.001
The last time you went to see a health professional, did you understand everything you were told?	No	614	24 (149)	8368	14 (1195)	1.83	1.50, 2.23	< 0.001
The last time you went to see a health professional, did you feel you were listened to?	No	606	14 (82)	8325	6 (466)	2.58	2.00, 3.33	< 0.001
Do you know how to contact the school nurse?	No	619	44 (271)	8424	35 (2929)	1.49	1.26, 1.76	< 0.001
Do you know about the school nurse drop in?	No	617	42 (260)	8393	43 (3604)	1.04	0.87, 1.23	< 0.001
Would you like an appointment to speak to the nurse about your health and how you feel?	Yes	606	14 (85)	8349	4 (302)	4.30	3.30, 5.60	< 0.001

### Ethics and governance

Favourable ethical opinion for the secondary data analysis was granted by the University of Central Lancashire, Science, Technology, Engineering, Medicine and Health ethics committee (Reference: STEMH864). It was asserted that most 13–14-year-olds would be competent to decide whether to participate or not, if provided with sufficient appropriate information. Based on Articles of the UN Convention on the Rights of the Child it was concluded that Year 9 pupils were the primary rights-holders over that decision and that parental consent for survey participation was not sought. Therefore, pupils determined their own participation, with the exception of pupils who required additional attention from school nurses to gain competence, or parental consent where competence could not be achieved.

## Results

Results are presented in line with our hypothesis that CYP in Lancashire affected by domestic and/or relationship violence will report greater levels of mental health, physical health and health risk vulnerabilities.

### Response rate and prevalence

A total of 9626 Year 9 pupils responded equating to 71% of eligible Year 9 pupils. Of the 9626 responses received, 9273 provided information for the domestic and/or relationship violence variable with 353 missing values. In total, 4623 participants were identified as male, 4527 as female and 26 as other; 628 (6.8%) pupils reported that they had been affected by domestic and/or relationship violence and all chi-squared tests showed significance. Table 1 shows demographic frequencies for pupils affected by domestic and/or relationship violence.

There were prevalence differences across demographic variables of gender, gender identity, ethnicity and sexual identity of being affected by domestic and/or relationship violence. More girls than boys, 7.8% and 5.6%, respectively, reported being affected. However, the highest prevalence (15.4%) was reported by CYP who did not identify as either male or female. Gender identity and sexual minority CYP (other gender, 15.4%; non-cisgender, 13.3%; gay/lesbian, 11.7%; bisexual, 21.1%; other [not straight], 15.3%) reported statistically significant higher rates of being affected than their cisgender (6.5%) and straight (6.2%) peers (Table 1). Differences between sexual majority and sexual minority CYP from this population have been analysed and published (Clarke et al. 2018). Across ethnic identities, CYP who identified as Asian or Asian British had the lowest reported prevalence rate (4.5%) followed by students identifying as White (7.4%), Mixed (9%) and Black or Black British (10.3%).

CYP who reported being affected by domestic and/or relationship violence had significantly poorer health and well-being across multiple domains in comparison with CYP not affected (Table 2).

### Mental health

CYP affected by domestic and/or relationship violence report poorer mental health states than non-affected CYP. CYP affected by domestic and/or relationship violence were six times more likely to not feel happy (OR 6.02; 95% CI: 4.88–7.46) and nearly five times more likely to feel lonely (OR 4.85; 95% CI: 4.03–5.85). They were also more likely to feel angry (OR 3.50; 95% CI: 2.92–4.20), less hopeful about their future (OR 3.02; 95% CI: 2.50–3.65) and to have negative perceptions about their body weight (OR 2.22; 95% CI: 1.87–2.63). Affected CYP were also nearly six times (OR 5.88; 95% CI: 4.88–7.09) more likely to undertake acts of deliberate self-harm.

Greater experience of bullying, as victim or perpetrator, was associated with being affected by domestic and/or relationship violence. Affected CYP were four times more likely to experience cyber-bullying (OR 4.00; 95% CI: 3.30–4.83), more likely to have been bullied (OR 3.76; 95% CI: 3.10–4.57) or taken part in bullying (OR 3.8; 95% CI: 2.81–5.15) and were less likely to know how to get help about bullying (OR 2.13; 95% CI: 1.64–2.78).

### Physical health

CYP affected by domestic and/or relationship violence reported poorer general health and well-being states. More reported having enduring health conditions that needed regular health support (OR 1.65; 95% CI: 1.35–2.01) and poorer oral care and diet; they were less likely to brush their teeth twice daily (OR 2.19; 95% CI: 1.71–2.79) and have regular dental care (OR 2.48; 95% CI: 1.72–3.56). They reported not eating as regularly (breakfast, dinner and tea (evening meal) the previous day (OR 2.53; 95% CI: 2.13–3.01) nor to have eaten any fruit or vegetables in the same period (OR 1.51; 95% CI: 1.23–1.86).

Affected CYP reported potentially harmful lifestyle behaviours, reporting greater frequency of consuming sugary drinks (OR 1.51; 95% CI: 1.27–1.80), more sedentary time watching television or gaming (OR 1.53; 95% CI: 1.28–1.82) and were more likely to have been drunk (OR 3.52; 95% CI: 2.87–4.31), smoked cigarettes (OR 5.56; 95% CI: 4.48–6.85), e-cigarettes (OR 4.15; 95% CI: 3.44–4.98) or shisha (OR 3.27; 95% CI: 2.42–4.42).

### Vulnerability

Affected CYP were at greater risk of exposure to drug cultures. They were more likely to be worried about drug use in school (OR 3.98; 95% CI: 3.22–4.95), worried about drug use by someone they know (OR 1.88; 95% CI: 1.38–2.54) and were five times (OR 5.49; 95% CI: 4.50–6.71) more likely to have been asked to try drugs than non-affected peers. Affected CYP were also nearly twice as likely to not feel confident to reject someone wanting physical or intimate contact (OR 1.96; 95% CI: 1.54–2.49). They were more likely to report having insufficient knowledge to prevent pregnancy (OR 1.40; 95% CI: 1.13–1.74) or sex-related infections (OR 1.27; 95% CI: 1.05–1.53). Affected CYP were also more likely to report having familial caring responsibilities (OR 2.18; 95% CI: 1.78–2.69).

Affected CYP were less likely to know how to get health information (OR 1.63; 95% CI: 1.32–2.01) and less likely to have an adult to talk to about problems (OR 3.23; 95% CI: 2.55–4.08) and were over four times more likely to request an appointment to see a school nurse following the survey (OR 4.3; 95% C.I.: 3.30–5.60). Compounding their poorer access to health resources, CYP affected by domestic and/or relationship violence reported poorer experiences of health care. They were more likely to not feel listened to (OR 2.58; 95% CI: 2.00–3.33), not receive enough information (OR 2.69; 95% CI: 2.13–3.40) and not understand everything they were told (OR 1.83; 95% CI: 1.50–2.23).

## Discussion

The results of this study showed the extent of associations found between exposure to domestic violence and/or relationship violence and CYP’s poorer health and well-being across multiple areas of life. Crucially, the sample in this study is large and representative of the study population, with CYP from urban and rural environments that include some of the most deprived and the most affluent in the UK.

### Prevalence of domestic and/or relationship violence

Seven per cent of pupils aged between 13 and 14 years old reported being affected by domestic and/or relationship violence. This was lower than in most violence surveys, possibly because of the framing of our question of ‘being affected’ by domestic and/or relationship violence rather than exposure alone. It is possible that CYP in our sample were exposed to this violence but did not feel affected by it. In this context, odds ratios reflect experiences of CYP who reported that domestic and/or relationship violence had an effect on their lives and the magnitude of the odds ratios across all domains are stark. Minority CYP, whether based on sexual, gender or ethnic identity, reported higher rates of being affected and likely signifies the effects of intersectionality in terms of axes of privilege, discrimination and inequalities.

### Mental health

CYP reporting being affected by domestic and/or relationship violence shared similar mental health experiences as peers in the USA (Mrug and Windle 2010) and Iceland (Gunnlaugsson et al. 2011). Witnessing adult violence at home was a predictor for anxiety, and victimisation by an adult at home was a predictor of depression for CYP (Mrug and Windle 2010). The study did not ask about suicidal ideation, though it did ask about deliberate self-harm, which was nearly six times greater amongst affected CYP. In common with studies in Sweden (Lucas et al. 2016) and China (Chen et al. 2018), affected CYP were much more likely to be involved in bullying including cyber-bullying as victim or perpetrator. Little is stated in the literature about loneliness and lack of future optimism in contexts of violence, which in this study were four and three times more likely to be reported by CYP affected by violence.

### Physical health

CYP affected by domestic and/or relationship violence were more likely to have enduring health problems requiring regular health care. In the USA, girls involved in dating violence as perpetrator or victim were more likely to report physical complaints than non-involved peers (Haynie et al. 2013). CYP in Denmark who were victims of violence at home or who witnessed violence against their mother at home were up to six times more likely to report poor health (Helweg-Larson et al. 2011). The category of ‘enduring health problem’ in this present study was broad as the survey asks several questions collapsed into one (‘Do you have any illness, conditions, or attend any regular hospital clinics, have regular health support or take regular medicine?’). CYP’s report of regular health intervention represents an array of mental or physical health needs, and associating this with violence exposure may appear tenuous. However, it is an important finding as more is understood about psychological and biological responses to acute and prolonged stressors as mediating pathways in foetal health and child development, and mental health and physical health conditions in contexts of violence (WHO 2013).

Affected CYP had greater negative perceptions of their body weight. This study provides evidence of mechanisms that may explain why these CYP had altered weight patterns, finding that affected CYP had less regular eating patterns, consumed less vegetables or fruit, consumed more sugary drinks and spent more time doing sedentary activities than non-affected peers. That said, these eating and activity habits, along with the finding of greater risk of poor oral health, could indicate greater health vulnerability risks connected with sub-optimal care-giving environments, as a consequence of domestic violence for primary caregivers. Sub-optimal care-giving may signal an erosion of parenting skills and indicate need for supportive intervention (Rizo et al. 2016).

### Vulnerability

Affected CYP in this study faced a greater range of vulnerability risks; some that concur with the existing literature and some that extend our understanding. Associations between dating violence and substance use and smoking are known (Haynie et al. 2013). Actual drug use by CYP was not surveyed; however, affected CYP had significantly higher levels of exposure to drug culture than their peers, with more having been asked to try drugs, worried about drug use at their school or by someone they knew. In congruence with an association between CYP’s exposure to violence and lower self-esteem (Gunnlaugsson et al. 2011), affected CYP in this study reported less confidence to reject unwanted physical or intimate contact, which may indicate greater vulnerability for sexual violence and/or exploitation.

CYP affected by domestic and/or relationship violence were more than twice as likely to have carer responsibilities, perhaps reflecting consequences of domestic violence on primary caregiver health (Ehrensaft et al. 2006; Trevillion et al. 2012) and/or family functioning (Rizo et al. 2016). The greater levels of loneliness reported by affected CYP may relate to isolation as a consequence of carer responsibilities, or of controlling behaviours experienced in relationship violence.

### Access to care and trusted adult

Compounding mental and physical health and vulnerability risks, CYP affected by domestic and/or relationship violence in this study were less likely to have an adult they could talk to or know how to get professional help. A good relationship with an adult is one factor that can moderate and protect against harms associated with exposure to violence (Kassis et al. 2013). However, relationships between protective factors and better outcomes are not straightforward. A study across four EU countries (Kassis et al. 2013) found that as levels of violence escalate, effects of protective factors diminish and CYP’s internal and external protective resources can be overwhelmed. Importantly, this study reporting on an ‘affected population’ illustrates multiple and potentially cumulative adversities in terms of intersectionality, health risks and vulnerabilities in comparison with non-affected peers, leading to widening trajectory of health inequality.

Moreover, this study found greater levels of negative health care experiences for CYP affected by domestic and/or relationship violence. Why is not clear, but it may result from professionals’ assumptions, attitudes and unintended communication barriers (Schnierle et al. 2019). Asked whether they would like an appointment with the school nurse, affected CYP in this study were four times more likely to say yes, a finding strongly indicative of a health access knowledge deficit. Lack of access to health information and poor access knowledge of affected CYP was a recurring theme across the data.

### Limitations

We were not able to fully control school environments during survey completion. Some schools afforded greater support than others which may have resulted in under-reporting.

Data were collected via an existing health needs assessment process, limited by operational factors, leading to quantitative variables that constrain more complex analysis. The large sample may result in more statistically significant results, of which some may have disappeared with advanced analysis. The addition of qualitative methods would have allowed greater understanding of pupils’ experiences.

The primary purpose of the survey necessitated a collapsing of domestic and relationship violence into one question, as both would result in similar response from school nursing services. This and the phrasing of the question may have contributed to under-reporting.

Pupils may have differing levels of understanding and health literacy, leading to variance in how questions are answered and demographic factors such as locality and school could not be adjusted for.

Ethnic diversity amongst participants was slightly greater than in Lancashire (LCC 2012) and England and Wales (ONS 2018), and whilst this sample is diverse, young black people are underrepresented in comparison with England-wide populations.

### Conclusion

This study provided an opportunity to analyse and better understand the associations and risks for mental and physical health and vulnerability of CYP affected by domestic and/or relationship violence. The magnitude of associations found between exposure and CYP’s poorer health and well-being across multiple domains makes an important contribution to academic and professional fields in the UK and globally. Whilst the findings support previous evidence, they also demonstrate significant and marked disparities across a wider panorama of health risk indicators and vulnerability, and poorer experiences of and access to support by health services. This double jeopardy of poorer health service access *and* poorer health service experience presents important learning for health and other services and an ethical obligation to improve the awareness and practice of health professionals, particularly those delivering school-age health and community outreach services. The health inequalities reported by CYP in this study provide compelling, contemporary intelligence for renewed strategic policy-level consideration in the design, interdisciplinary pathways and delivery of young peoples’ health and other services in order to better identify, respond and be trusted by CYP.
